# Insight into others’ minds: spatio-temporal representations by intrinsic frame of reference

**DOI:** 10.3389/fnhum.2014.00058

**Published:** 2014-02-14

**Authors:** Yanlong Sun, Hongbin Wang

**Affiliations:** ^1^The University of Texas Health Science Center at HoustonHouston, TX, USA; ^2^Center for Biomedical Informatics, Texas A&M University Health Science UniversityHouston, TX, USA

**Keywords:** theory of mind, false belief, spatial cognition, frame of reference, predictive learning

## Abstract

Recent research has seen a growing interest in connections between domains of spatial and social cognition. Much evidence indicates that processes of representing space in distinct frames of reference (FOR) contribute to basic spatial abilities as well as sophisticated social abilities such as tracking other’s intention and belief. Argument remains, however, that belief reasoning in social domain requires an innately dedicated system and cannot be reduced to low-level encoding of spatial relationships. Here we offer an integrated account advocating the critical roles of spatial representations in intrinsic frame of reference. By re-examining the results from a spatial task (Tamborello etal., 2012) and a false-belief task (Onishi and Baillargeon, 2005), we argue that spatial and social abilities share a common origin at the level of spatio-temporal association and predictive learning, where multiple FOR-based representations provide the basic building blocks for efficient and flexible partitioning of the environmental statistics. We also discuss neuroscience evidence supporting these mechanisms. We conclude that FOR-based representations may bridge the conceptual as well as the implementation gaps between the burgeoning fields of social and spatial cognition.

## INTRODUCTION

Recent research has seen a growing interest in the connections between two disparate lines of investigations: spatial cognition that focuses on spatial and bodily representations, and, social cognition that examines the abilities of attributing other’s intentions and beliefs, namely, theory of mind (TOM). Although researchers have learned much about the underlying mechanisms in each domain, there are still opposing perspectives and considerable conceptual gaps between the two domains. In particular, much contest revolves around the contribution of domain-specific spatial processing to domain-general TOM abilities.

At the center of the debate, is an apparent contradiction between the findings that human infants can pass false-belief tasks (e.g., holding an agent’s belief about the original location of an object, which has been changed in the absence of the agent) and the general claim that children first understand false-beliefs at around 4 years of age (for reviews, see, Apperly and Butterfill, 2009; Perner et al., 2011; Frith and Frith, 2012). Some have suggested that sophisticated TOM inferences, as indicated by successfully performing the false-belief tasks, may evolve from a set of low-level encoding processes, for example, agent-object-location associations (Perner and Ruffman, 2005; Ruffman and Perner, 2005), identification of “external referent” (Perner et al., 2011), and, spatial perspective taking (Kessler and Rutherford, 2010; Kessler and Thomson, 2010). Yet other theorists have posited that beliefs are “invisible abstract entities” (Saxe, 2006), and that making inferences about other’s beliefs requires a dedicated or innate system that cannot be accounted for by mere associations (Leslie, 2005; Saxe and Wexler, 2005; Csibra and Southgate, 2006; Baillargeon et al., 2010).

In the present paper, we attempt to bridge the conceptual gaps between different perspectives by advocating an integrated account. We argue that a fundamental spatio-temporal association process, which is fraught in the domain of spatial cognition, is also essential in the domain of social cognition. At the computational level, spatio-temporal association is to extract statistical regularities from the task environment by detecting the correlations between representations of events over space and time. However, spatio-temporal association is not merely about matrices of associative weights that connect different representations in a static manner. Instead, it takes place over space and time through the lens of *predictive learning*. Recent advances in neuroscience suggest that – at both the algorithmic and neural architectural levels – it is not reward that drives learning *per se*, but the temporal discrepancy between actual and expected outcomes (Gerstner et al., 2012; O’Reilly et al., 2012). That is, the task environment constantly changes. At any moment, environmental statistics present themselves as multimodal inputs to the mind. By constantly comparing the observed and expected outcomes, the mind selectively re-encodes the raw environmental statistics and transforms them into a hierarchy of representations at different levels of abstraction, which eventually produce complex behaviors such as thought, language, and, intelligence (Hawkins and Blakeslee, 2004).

Our approach to understanding the process of spatio-temporal association utilizes frames of reference (FOR) as the building blocks of both spatial and social cognition. A growing body of research has shown that FOR-based representations are not only behaviorally plausible but are also supported by the neurological structures in both human and animal brains. As spatio-temporal association re-encodes the environmental statistics by removing task-irrelevant variances (e.g., instability, noise), FOR-based representations provide a straightforward way of partitioning spatio-temporal variances. In addition, it has been a central contention that theory-of-mind abilities are subject to competing demands for efficient and flexible processing and require two distinct systems, “one that is efficient and inflexible and one that is flexible but cognitively demanding” (Apperly and Butterfill, 2009, p. 957). Instead of focusing on the distinction between different systems, we emphasize the common representations shared by different sets of abilities and mechanisms. We argue that when people perform spatial and social tasks, both efficiency and flexibility can emerge from the expectation-driven competition among multiple FOR-based representations.

## INTRINSIC FRAME OF REFERENCE (IFOR) IN SPATIAL COGNITION

The notion of “FOR” has been crucial to all the disciplines that study spatial relationships and relies on a diverse terminology for its classification (Levinson, 2004). For example, a conventional approach is to classify a reference system by its origin: whether it is anchored to the observer self (e.g., “egocentric”) or the environment (e.g., “allocentric”; Andersen et al., 1997; Wang and Spelke, 2002; Burgess, 2008). However, we adopt a classification system that – besides the self-centered egocentric frame of reference (EFOR) – further differentiates the environment-centric frames into two categories: *allocentric* (AFOR, with an absolute and fixed anchor), and, *intrinsic* (IFOR, with a relative and flexible anchor). With roots in psycholinguistic research, the advantage of this classification scheme is that it reduces ambiguity in spatial descriptions of the world (Miller and Johnson-Laird, 1976; Carlson-Radvansky and Irwin, 1993; Levelt, 1996; Levinson, 2004; Carlson and Van Deman, 2008). For example, when describing the location of a coffee cup, one may say, “the cup is in front of me (observer self)” (in EFOR); “the cup is on the desk” (in AFOR); or “the cup is in front of John” (in IFOR). Note that, while both AFOR and IFOR use an external anchor, the anchor in AFOR (the desk in this case) is more stable than IFOR (John in this case, who can freely change his location or orientation). Our interest in IFOR is motivated by vision and spatial memory research that emphasizes the dynamic updating of object-centered representations (Marr, 1982; Wang et al., 2005a; Mou et al., 2008; Sun and Wang, 2010; Chen and McNamara, 2011). In this respect, the interactions between EFOR and IFOR (e.g., the intertwined representations of self-other-object relationship) are ubiquitous in everyday tasks, where the “other” can be either an anchoring object (Wang et al., 2005b; Tamborello et al., 2012), or another agent or human being as in social situations (Mitchell, 2006; Kessler and Rutherford, 2010; Kessler and Thomson, 2010; Perner et al., 2011).

One fundamental distinction among different FOR-based representations is the manner in which each representation handles *temporal instability* during the interactions between the mind and the environment. Temporal instability manifests itself as both spatial and temporal variances during the encoding of spatio-temporal relationships between various entities in the environment (e.g., self, agents, objects, locations, and events). Different reference systems partition these variances in different manners and therefore afford structures at different levels of instability. In the “coffee cup” example, the spatial relations among relevant entities can change over time. To locate the coffee cup, an EFOR representation from the observer’s perspective is relatively stable, to the extent that the anchor is always the “observer self.” In contrast, an IFOR representation of the coffee cup anchored to John is unstable because John can freely move around and the observer is therefore required to track both the coffee cup and John in order to maintain an IFOR representation.

Critically, temporal instability evokes *predictive learning. *Simply put, whereas temporal instability means that the current input is expected to change at the next time point, predictive learning is a process of spatio-temporal integration in which the internal representation is constructed by remapping attention toward the expected outcomes (Hawkins and Blakeslee, 2004; O’Reilly et al., 2012). It has been suggested that predictive learning is a driving force in learning structured abstractions of the environment (Hawkins and Blakeslee, 2004; Krauzlis and Nummela, 2011; Rolfs et al., 2011; Gerstner et al., 2012; O’Reilly et al., 2012). Consider the coffee cup example again: predictive learning takes the anticipated movements into consideration and produces a dynamic representation of the relevant spatial relations. When an observer is reaching for a coffee cup, predictive learning occurs within EFORs, such that the coffee cup’s location is updated relative to the observer’s hand or body. By making constant predictions, the observer would know when to grab even before her hand touches the cup. When the observer watches John reaching for the coffee cup, predictive learning involves IFORs, such that the coffee cup’s location is updated relative to John. Yet, should John suddenly change his course and pick up another object (e.g., a stapler), the observer would be surprised as John’s initial movements led to an expectation that he would pick up the coffee cup instead of the stapler.

**FIGURE 1:**
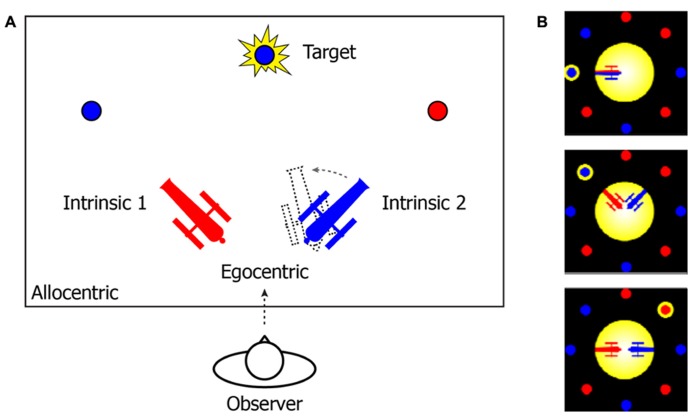
**(A)** A schematic illustration of the two-cannon task reported by Tamborello et al. (2012). In the experiment, participants (“observers”) were sitting in front of a computer (the observer’s line of sight was perpendicular to the plane of the computer screen). **(B)** Actual task displays in the experiment. At the beginning of each trial, two cannons (one red and one blue) and multiple pellets (in either red or blue) were presented together on the computer screen. After a one-second pause, a randomly selected pellet would flash as the target. The participants’ task was to use the arrow keys to rotate the cannon in the same color of the target toward the target as quickly as possible. The cannons’ orientations and the ratio between the number of red and blue pellets were varied across trials. The angles between two cannons were either “conflict-absent” (zero degree) or “conflict-present” (90 or 180°).

That the mind uses different FOR to manage temporal instability and drive spatio-temporal association is consistent with an accumulating body of neurological and behavioral studies (Marr, 1982; Krauzlis and Nummela, 2011; Pertzov et al., 2011; Rolfs et al., 2011; Van Der Werf et al., 2013). To further illustrate this notion, consider an example from the two-cannon experiment reported by Tamborello et al. (2012). In their experiment (**Figure 1**), participants were instructed to use the arrow keys to rotate the cannon in the same color of a to-be-revealed target as quickly as possible, so that the cannon could point to (and shoot at) the target. Three different types of reference systems can be used to describe the target location (**Figure 1A**). In an EFOR representation (relative to the observer), the target is at the front-top of the observer’s visual field (the observer’s line of sight was perpendicular to the plane of the computer screen). In an AFOR representation, the target can be described in reference to the computer screen frames. In an IFOR representation (relative to a cannon), the target has a counterclockwise bearing relative to the orientation of the blue cannon (or a clockwise bearing relative to the red cannon). Mathematically, all of these representations are equivalent, to the extent that one representation can be transformed into another without losing any information. However, in terms of efficient and flexible removal of task-irrelevant variance, different representations are unique in the way they are updated and maintained.

**FIGURE 2:**
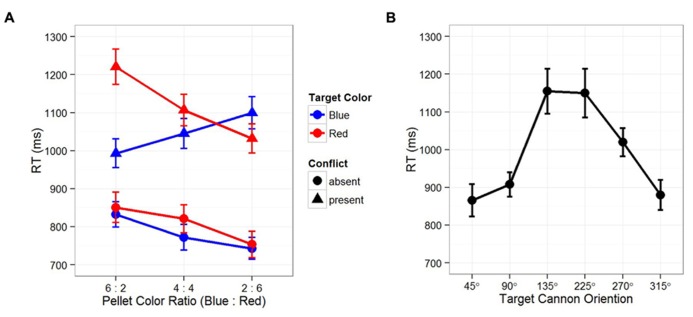
**Results from Tamborello et al. (2012). (A)** Reaction times as function of target color, conflict between two cannons, and the color ratio in surrounding pellets. Across three sequentially presented blocks of trials, the surrounding pellets varied from trials of more blue pellets (B:R = 6:2) to trials of more red pellets (B:R = 2:6). Reaction times in the “conflict-absent” conditions (two cannons pointing to the same direction) were significantly faster than that those in the “conflict-present” conditions (90 or 180° between two cannons). Within the “conflict-present” conditions, reaction times were significantly faster for the cannon in the same color of the majority pellets, indicating the effect of expectation, where participants had made predictions on the task-relevant cannon before the appearance of the actual target. **(B)** Reaction time was dependent on the angular disparity between the participants’ “up” and the target cannon orientations (self-cannon variance), indicating an interaction between EFOR and IFOR representations, namely, the effect of perspective taking. Note that since participants were always facing the computer screen, their “up” was congruent with the “up” on the computer screen. In both figures, error bars depict standard error of the mean.

Let us first examine temporal instability. It is clear that both EFOR and AFOR representations have relatively fixed anchors (e.g., the observer and the computer monitor frames, respectively). In contrast, IFOR is only *tentatively* anchored to one of the two cannons: the color and location of the target is initially unknown, thus, which cannon is task-relevant depends on the visual input at the next time point. Recall that temporal instability evokes predictive learning, in which internal representations of the environment are constructed based on the current observations toward the expected future outcomes. In this case, the color ratio of the pellets provides a reliable cue for predicting the relevancy between two competing cannons. **Figure 2A** shows that reaction times in the conflict-present condition (cannons pointing to different directions) were significantly slower than those in the conflict-absent condition (cannons pointing to the same direction). Within the conflict-present conditions, the cannon in the same color of the majority pellets resulted in faster reaction times. These results indicate that in resolving the conflict between different IFOR representations, participants planned their responses by predicting the task-relevant cannon based on the pellet color ratio. That is, prediction occurs before the appearance of an actual target, leading to a stronger IFOR representation anchored to the task-relevant cannon, thus resulting in faster reaction times.

Second, in order to achieve computational efficiency and flexibility, multiple IFOR representations may coexist and interact with each other. **Figure 2A** shows that even when participants made correct predictions on the task-relevant cannon in the conflict-present condition, their reaction times were still significantly slower than that in the conflict-absent condition. This indicates that, while anticipating the upcoming target, the competition between two conflicting IFOR representations resulted in a partial dissociation. That is, as the IFOR representation anchored to the predicted task-relevant cannon was the focus of attention, the other one was only partially disengaged – a strategy of prioritizing but still preparing for the unexpected. As a result, even when the prediction was correct, the partially disengaged IFOR representation interfered with performance and produce longer reaction times.

Third, an interaction may also occur between EFOR and IFOR representations. **Figure 2B** shows that reaction times were significantly dependent on the angular disparity between the self and cannon orientations, indicating a strategy of combining EFOR and IFOR representations, or *perspective taking*. Perspective taking has been considered as an important stepping stone from automatic and unaware perception toward a conscious and deliberate process in which people mentally perform a movement simulation of other people or objects (Kessler and Rutherford, 2010; Kessler and Thomson, 2010; Zwickel et al., 2011). Here, we consider perspective taking in terms of partitioning the statistical variances in the task environment.

Specifically, for a given cannon, we consider three parts of the spatial variances (angular disparities) that could be mentally encoded: self-cannon, self-target, and cannon-target. Since the correct response is determined by the cannon-target variance, it requires either a complete or a partial disengagement of the EFOR representation. If the EFOR representation is to be completely disengaged (i.e., removing self-target and self-cannon variances), the task could be accomplished by *object rotation* based only on an IFOR representation. However, the reaction time pattern in **Figure 1B** suggests a case of partial EFOR disengagement: the task was accomplished by *self rotation with perspective taking*, in which the self-cannon variance was first removed so that the self-target variance became exactly the same as the cannon-target variance. Similar to the interaction between multiple IFOR representations, the interaction between EFOR and IFOR representations also serves the purpose of both computational efficiency and flexibility. On the one hand, an IFOR representation is parsimonious in encoding only task-specific variances (e.g., encoding only the target-cannon but not the self-cannon, the self-target relations). On the other hand, an EFOR representation tend to be automatic and effortless (Wang and Spelke, 2002; Frith and Frith, 2007; Kessler and Thomson, 2010). Therefore, an efficient and flexible solution would be to combine EFOR and IFOR representations into one representation. That is, instead of utilizing a purely IFOR-based strategy in which the cannon is mentally rotated toward the target (i.e., object rotation), participants might superimpose their egocentric perspective onto the cannon – that is, take the perspective of the cannon – then mentally self-rotate toward the target.

Overall, this new interpretation of the two-cannon experiment results suggests that expectation-driven competitions can take place not only between different IFOR representations (**Figure 2A**), but also between EFOR and IFOR representations (**Figure 2B**). By this account, the internal spatial representation of the environment is always dynamically constructed and updated toward the anticipated outcomes, rather than static associations of the current spatial configuration. Depending on whether there are conflicts between representations and whether the actual outcome meets the expectation, competition takes place at different levels and results in the engagement and disengagement of different FOR-based representations. In the following section, we demonstrate that the same mechanisms may well lay the foundation for more complex representations in the domain of social cognition.

## INTRINSIC FRAME OF REFERENCE IN BELIEF ATTRIBUTION

A landmark finding in belief attribution is that fifteen-month-old infants appear to be able to appeal to other’s beliefs, that is, they were able to keep track of an actor’s perception about the location of a toy, and, using this perception rather their own, to predict the actor’s searching behavior (Onishi and Baillargeon, 2005). This finding has triggered a substantial debate over the question whether the theory-of-mind abilities evolved from “actor-object-location associations” (Perner and Ruffman, 2005, p. 215), or are due to an innate mechanism specialized for belief attribution (Leslie, 2005; Baillargeon et al., 2010). Here we offer a reinterpretation of the original findings based on the same spatio-temporal association account outlined above.

**FIGURE 3:**
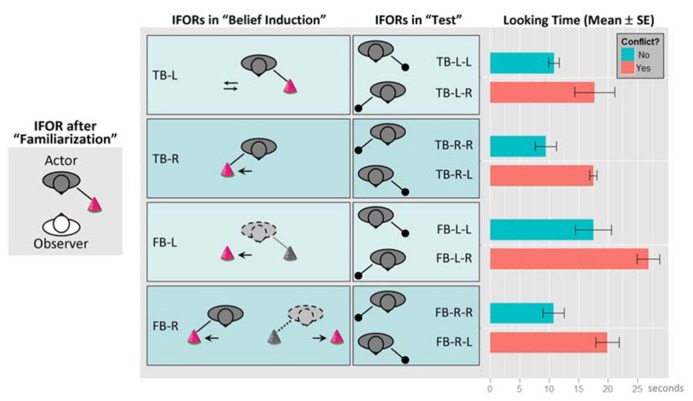
**The experimental setup and results, re-produced based on Onishi and Baillargeon (2005).** Conditions have been renamed by replacing the original labels “green box” and “yellow box” with location labels “L” and “R”, respectively (“L” and “R” indicate the toy’s location from the actor’s perspective). The experiment consisted of three phases: (1) “familiarization”, (2) “belief induction”, and (3) “test”. During (1), infants (“observer”) watched the actor reaching toward a box for a toy at one of two locations (boxes are not shown here). At the end of this phase, the toy was located on the actor’s left-hand side. In (2), infants were assigned to one of four conditions, in which they watched some movements of the boxes or the toy in the actor’s presence or absence. Here we used dyadic labels to represent the validity of the actor’s belief (“TB” for true belief and “FB” for false-belief) and the location of the toy last known to the actor from the actor’s perspective (“L” for the left-hand side and “R” for the right-hand side). In addition, arrows represent movements of the box or the toy; colored toy and solid lines indicate the actor’s true belief about the toy’s location; grayed toy and dotted lines represent the actor’s false-belief as the location of the toy was changed in her absence. In the “TB-L” condition, the toy remained at the actor’s left-hand side and only the box at the actor’s right-hand side was moved toward the toy then back to its original location. In the “TB-R” condition, the toy was moved from the actor’s left to her right in her presence. In the “FB-L” condition, the toy was last seen by the actor at her left but was moved to her right in her absence. In the “FB-R” condition, the toy was moved from the actor’s left to her right in her presence but moved back to her left in her absence. In test phase (3), infants watched the actor reaching one of the locations for the toy and their average looking times were recorded and analyzed. Here we use triadic labels to represent each test condition, with the first two parts repeating the label for the corresponding belief induction condition, and the last part representing the direction where the actor reached for the toy. The equality between the last two parts represents whether there is a conflict between the IFOR representation at the end of the belief induction phase and the one in the test phase. For example, “TB-L-L” represents the condition in which the actor held a true belief that the toy was at her left-hand side and she actually reached the same location for the toy (“no conflict”); In comparison, “TB-L-R” represents the condition in which the actor held a true belief that the toy was at her left-hand side but she actually reached her right-hand side for the toy (“conflict”). Infants’ looking times (mean and standard errors in seconds) in each test condition are shown on the rightmost panel.

**Figure 3** re-produces the experimental setup and results from Onishi and Baillargeon (2005). Note that we have re-labeled the experimental conditions by replacing the original object labels with location labels from the actor’s perspectives: “green box” replaced by “L” (actor’s left-hand side), and, “yellow box” replaced by “R” (actor’s right-hand side). Hence, our new labels are essentially placeholders for representing different locations. However, the new labels also highlight the spatial component of the task environment and potential interference between the different FOR. Similar to the two-cannon experiment, this task involves the interplay of multiple representations. For example, the toy’s location can be described in EFOR (relative to the observer, which is the infant in the experiment), AFOR (relative to the table or the room), or IFOR (relative to the actor). According to the original object labels, the toy’s location was described by the color of the box, which was the same to both the infant and the actor. In contrast, as the infant was facing the actor, the “left” and “right” labels were completely opposite, depending on whether they were from the infant’s perspective (EFOR) or from the actor’s perspective (IFOR). Therefore, the new labels were more effective in distinguishing EFOR and IFOR representations.

### COMPARISON WITHIN BELIEF INDUCTION CONDITIONS

The main finding by Onishi and Baillargeon (2005) involved comparing the infants’ looking times between the two “test” conditions within each of the four “belief induction” conditions. They reported that looking times were shorter when the actor reached for the toy where she believed it was located (“no conflict” conditions in **Figure 3**) and longer when the actor reached the opposite location (“conflict” conditions). Based on this comparison, the authors concluded that infants were able to use the actor’s belief state instead of the actual toy location from infants’ own perspective to predict the actor’s reaching behavior.

Rather than resorting to an innately dedicated belief attribution mechanism, we would like to offer a different explanation based on fundamental spatial information processing mechanisms. Our interpretations is that belief attribution derives from the proper maintenance of and dissociation between multiple representations based on EFOR (for encoding self-toy or self-actor relations) and IFOR (for encoding actor-toy relations). In particular, it has been suggested that infants’ looking time provides a measurement of surprise, such that longer looking times indicate greater violation of infants’ expectations relative to their prior knowledge or greater novelty relative to their interpretation of habituation stimuli (Baillargeon, 1986; Onishi and Baillargeon, 2005; Téglás et al., 2011). Here we argue that for the false-belief task by Onishi and Baillargeon (2005) surprise might have resulted from the violation of infant’s expected spatial configuration relative to the actual one. Our earlier argument suggests that, among all possible FOR-based representations, those leading to task-relevant predictions tend to be actively updated and maintained. Since the looking times were about the actor’s reaching for the toy, both the expected and actual spatial configurations would be encoded in the form of IFOR representations (actor-toy), rather than irrelevant EFOR representations (infant-toy). In other words, the IFOR-based expectation reflects a simple behavioral rule by means of spatial association – people (the actor) look for objects at their last known location (Ruffman and Perner, 2005). Consequently, the difference in looking times between “conflict” and “no conflict” conditions may be explained by the effort of resolving the discrepancy between the IFOR representation at the end of the belief induction phase, relative to the actual IFOR representation in the test phase. Results in **Figure 3** support this explanation by showing that, in each of the four belief conditions, looking times were reliably longer (with a mean difference always around 7~9 s) when there was a conflict between the IFOR representations at the end of the induction phase (the same as the expectation) and in the test phase (the actual outcome). For example, looking times for “*x*-L-R” conditions were consistently longer than those for “*x*-L-L” conditions (“*x*” stands for either “TB” or “FB”, and, a conflict is present if the last two alphabets are different).

### COMPARISON BETWEEN BELIEF INDUCTION CONDITIONS

It is apparent from **Figure 3** that there were differences in looking times among the four belief induction conditions. For example, whereas the FB-L condition had the longest looking times, the FB-R condition had similar looking times as those in TB conditions. It is surprising that these differences were not mentioned nor accounted for by Onishi and Baillargeon (2005). Using the same argument in the two-cannon task, we speculate that the looking time difference between belief induction conditions might also be due to the interference from a partially disengaged representation. In this case, there could be different levels of the dissociation between EFOR and IFOR representations due to the different sequences of temporal events during the belief induction phase. Based on the comparison between “test” conditions above, it appears that the surprise effect (i.e., “conflict” versus “no conflict”) in all belief induction conditions remained approximately constant (7~9 s). This implies that the variance in looking times, less the surprise effect, would be independent of the predictions by the actor-toy IFOR representation. Accordingly, the remaining variance in looking times could be due solely to the interference from the infant-toy EFOR representation.

In the following, we use the conditional means and standard errors reported in the original study to make three sets of *post hoc* comparisons across different belief induction conditions but within the same “conflict” or “no conflict” test conditions (e.g., *x*-L-L compared with *x*-R-R, *x*-L-R compared with *x*-R-L, and etc.).

First, the mean looking times were about the same in the TB-L and TB-R conditions (i.e., TB-L-L ≈ TB-R-R, and, TB-L-R ≈ TB-R-L), despite different manipulation sequences in the belief induction phase – the former (TB-L) only involved the movement of an empty container (the “yellow box” on the actor’s left hand side) and the latter (TB-R) involved the change of the toy’s location (see **Figure 3**). This indicates that the looking times were primarily determined by the active maintenance of the IFOR representation of the actor-toy relationship. If there was any interference from the EFOR representation of the infant-toy relationship, the effect remained constant between these two conditions.

Second, the mean looking times were significantly longer in the FB-L condition than in the TB-R condition (i.e., FB-L-L > TB-R-R, mean difference ≈ 8 s; FB-L-R > TB-R-L, mean difference ≈ 9 s; two tailed *p* < 0.05 in both comparisons). Such differences could be accounted for by stronger interference from the EFOR representation in the FB-L condition than in the TB-R condition. Specifically, the change of the toy’s location was visible only to the infant in the FB-L condition but visible to both the infant and the actor in the TB-R condition. Thus, the infant-toy EFOR representation in the FB-L condition would be relatively stronger (more engaged). Being task-irrelevant (e.g., irrelevant to the actor’s fetching action), the stronger EFOR representation in the FB-L condition would lead to greater interference, resulting in longer looking times during the test phase.

Third, the mean looking times were significantly shorter in the FB-R condition than in the FB-L condition (i.e., FB-L-L > FB-R-R, mean difference ≈ 7 s; FB-L-R > FB-R-L, mean difference ≈ 7 s; one tailed *p* < 0.05 in both comparisons). Interestingly, despite the more complicated manipulation sequences in the FB-R condition, looking times were about the same as those in the true belief conditions (TB-L and TB-R). Consistent with the aforementioned explanation, it is likely that the IFOR representation in the FB-R condition became stronger when it was reinforced in the presence of the actor (the actor last saw the toy moving to her right-hand side). By competition, a stronger IFOR representation led to a weaker EFOR representation. Although both were false-belief conditions, the weaker EFOR representation in the FB-R condition resulted in less interference and, therefore, shorter looking times than the FB-L condition.

In summary, it appears that FOR-based representations may provide a more transparent and detailed explanation to the findings reported by Onishi and Baillargeon (2005). In contrast to the two-cannon experiment by Tamborello et al. (2012), this false-belief task was not explicitly designed to detect the EFOR–IFOR interaction (e.g., infants were always facing the actor with the same bearing). Therefore, the interpretation of our *post hoc* comparisons between belief induction conditions could be limited. Nevertheless, our interpretation remained consistent across all comparisons and across both tasks. That is, in order to track and predict other agent’s behavior, the internal process would involve at least a partial disengagement of EFOR representations, an active engagement of IFOR representations, and, potential interference between EFOR and IFOR representations.

Note that our interpretation is in the same vein as the “actor-object-location association” account (Perner and Ruffman, 2005). In addition, we identify the role of EFOR–IFOR dissociation. This interpretation is along the same line as the proposals that belief attribution may evolve from low-level spatial encoding processes, including the identification of “external referent” (Perner et al., 2011) and perspective taking (Kessler and Rutherford, 2010; Kessler and Thomson, 2010). Similar to the original interpretation by Onishi and Baillargeon (2005), here we also emphasize the role of expectation. However, expectation in our account is not the end product of belief attribution. Rather, it starts early at the level of FOR-based spatial representations. In this respect, belief representation emerges as the mind integrates different spatial representations at different time points by reducing the discrepancy between the actual and the expected outcomes.

## FROM SPATIAL TO SOCIAL: THE COMMON NON-COGNITIVE ORIGINS

Although we have demonstrated that the same language from spatial cognition may be used to interpret infants’ performance in the false-belief task, we do not claim that social cognitive abilities can be completely accounted for by those in spatial cognition. Moreover, we do not claim a parallel between an explicit spatial orientation task and 15-month-old infants’ preferential looking task. Rather, we focus on the common representations underlying these two seemingly different tasks. We argue that abilities from both spatial and social domains share common non-cognitive origins at the level of spatio-temporal association in extracting the environmental statistics. Ergo, these abilities, even if they appear different from each other, may not be domain-specific *per se*, but reflect the different requirements in computational efficiency and flexibility.

In bridging the conceptual gaps between spatial and social cognitive abilities, it is critical to understand the common dynamic nature of spatio-temporal association in both domains. In the present paper, we have shown that, in terms of FOR-based representations, the two-cannon task and the false-belief task share at least three computational properties. First, both tasks require encoding multiple spatial relations with different reference points (spatial association); Second, both involve comparisons of representations at different time points (temporal association); Third, the internal representations for both tasks are not static spatial encodings at isolated time points, rather, they are constructed and maintained through competitions toward the expected outcomes (predictive learning). We argue that all these three properties are governed by the same principle, whether one’s goal is to learn a spatial configuration or infer other’s intentions and beliefs. That is, the internal representations are developed in the direction of reducing spatio-temporal instability (variances) in order to extract statistical regularities at different levels of abstraction from the task environment.

Commonly shared computational processes could well be supported by commonly shared neural implementations. A growing body of research suggests that brain mechanisms supporting sophisticated social abilities may derive from low-level processes such as spatial tracking, predictive encoding, and attention shifting (for reviews, see, Mitchell, 2006; Corbetta et al., 2008; Frith and Frith, 2012). In the same vein, we argue that the key ingredient in both spatial and social cognition is the expectation-driven competition between multiple FOR-based representations, that are supported by a set of intrinsically distributed neural networks, rather than separately dedicated brain mechanisms. In the following, we discuss the neural evidence that supports this view.

Even a simple task could demand multiple representations of the task environment at different temporal points. Then, the need for selection arises at different levels of processing due to the limitation of resources. On the basis of functional and anatomical distinctions, a model of attention selection has been proposed, suggesting that the attentional operations are carried out by the interactions between two fronto-parietal systems – a dorsal attention system (also referred to as top-down attention network, or, canonical sensory-motor pathway) and a ventral attention system (or, bottom-up attention network; Corbetta and Shulman, 2002; Corbetta et al., 2008; Yeo et al., 2011). The dorsal system is bilateral and mainly composed of the frontal eye field (FEF) and the intraparietal sulcus (IPS). It is specialized for selecting and linking stimuli and responses by sending top-down “filtering” signals to visual areas and via the middle frontal gyrus (MFG) to the ventral network. The ventral system is right-lateralized and includes the right temporal-parietal junction (TPJ), the right ventral frontal cortex (VFC), parts of the MFG, and the inferior frontal gyrus (IFG). Coordinated by the dorsal system, the ventral system sends bottom-up “reorienting” signals that interrupt and reset ongoing activity upon detection of salient targets, especially when there is a violation of expectation (for reviews, see, Corbetta et al., 2008).

The filtering and reorienting functionality in the dorsal–ventral attention networks is particularly useful for implementing the computation of multiple FOR-based representations, particularly when multiple FORs compete. We consider two levels of competition: (1) competition within the dorsal pathway (filtering), and (2), competition carried out by the interaction between the dorsal and ventral pathway (reorienting). Some evidence suggest that, along the dorsal pathway, multiple representations in different FOR can coexist – from lower-level retinotopic representations to higher-level self-centered (EFOR) and world-centered representations (IFOR and AFOR), and that the parietal cortex, particularly the IPS, is central to the construction of these representations (Marr, 1982; Andersen et al., 1997; Colby and Goldberg, 1999; Burgess, 2008; Pertzov et al., 2011; Van Der Werf et al., 2013). Recent rest-state data indicate that the dorsal attention network follows a serial and hierarchical organization, whereas the functional connectivity of parietal and prefrontal association cortices appears to be embedded with largely parallel and interdigitated circuits (Yeo et al., 2011). We argue that such an organization would allow a hierarchical abstraction of the task environment based on flexible selections among multiple representations. That is, in terms of FOR-based representations, it is possible that the invariance extracted at early cortical stages (e.g., visual areas and the parietal cortex) is incomplete, causing different representations to overlap with one another. In order to support higher-level abstractions, a more complete dissociation is required at the level of the prefrontal areas. For instance, it has been suggested that the FEF region plays a crucial role in the construction of intrinsic reference frames among multiple objects in spatial tasks (Wallentin, 2012). Likewise, studies with neural network simulations have shown that, although partial dissociation between different types of spatial information can occur by re-encoding visual information in the parietal cortex, dorsal control from the prefrontal cortex is necessary to achieve a more explicit dissociation (Sun and Wang, 2013); Moreover, efficient and flexible representations of the changing environment requires the maintenance of both latent representations (through altered firing thresholds in non-frontal regions) and active representations (through sustained firing in the prefrontal cortex) (Morton and Munakata, 2002). It is suggested that such a maintenance mechanism is involved when the infants created actor-object-location associations in the false-belief task (Perner and Ruffman, 2005).

More dramatic competition between multiple representations would likely occur when expectations derived from actual sensory input have been violated. In such instances, the ventral attention network sends out reorienting signals and the dorsal attention network is reconfigured (Corbetta et al., 2008). Evidence for dorsal–ventral interaction comes from studies that use perspective taking tasks, which typically involve conflicting perspectives in EFOR and IFOR representations. For example, it has been reported that the transformation from participants’ own perspective to another agent’s body axis was associated with activations in posterior parietal cortical regions, such as the left inferior parietal lobe (IPL) and parietal–temporal–occipital junction as well as the right superior parietal lobe (Vogeley et al., 2004; David et al., 2006). Additionally, it has been found that TPJ shows enhanced activities in voluntary orienting of attention when participants are cued about the future location of a target stimulus (Corbetta et al., 2000), and when they need to distinguish between self-produced actions and actions generated by others (Blakemore and Frith, 2003; Jackson and Decety, 2004). Recently, Mazzarella et al. (2013) reported that responses in right IFG are sensitive to another person’s orientation when participants perform the task from their own egocentric perspective. Thus, these studies are consistent with the suggestion that taking another person’s perspective requires extra effort as compared with using one’s own perspective (Kessler and Thomson, 2010).

It should be pointed out that among different brain areas, the TPJ region has been a major topic of debate regarding the neural mechanisms of belief attribution abilities in social interactions. Some researchers argue that this region is specifically involved in the theory-of-mind functions (Saxe and Kanwisher, 2003; Apperly et al., 2004; Saxe and Wexler, 2005; Saxe and Powell, 2006; Saxe et al., 2009; Young et al., 2010). However, the studies mentioned above suggest that the TPJ’s function is not unique in the social context. In fact, many theorists consider the TPJ the key hub of the ventral attention network, which essentially supports attention reorienting for resolving conflicts between different visual perspectives, especially when there is a violation of the expected outcomes (Posner et al., 2006; Decety and Lamm, 2007; Mitchell, 2008; Perner and Aichhorn, 2008). Similarly, it has been suggested that the dorsal part of the TPJ region is involved in representing different perspectives and making behavioral predictions, whereas the more ventral part of TPJ and the medial prefrontal cortex region (MPFC) are responsible for predicting behavioral consequences (Aichhorn et al., 2006). Along the same line, Corbetta et al. (2008, p. 317) posited that, “Similar environmental and bodily representations and their comparison may be co-opted for ToM interactions and that attention signals in TPJ may be important to switch between internal, bodily, or self-perspective and external, environmental, or other’s viewpoint, a key ingredient of ToM.”

In sum, we argue that by supporting different levels of competition between multiple representations, the functions of dorsal–ventral attention networks play a major role in both spatial and social cognitive abilities. Whereas the filtering function manages competition among representations required for the ongoing activity, the reorienting function facilitates competition and reconfiguration when the new sensory input violates the expectation from the current representations. Crucially, different levels of competition allow partial engagement (or disengagement) of certain representations, which facilitate the integration of potentially conflicting representations. As mentioned earlier, maintaining multiple IFOR representations is essential for prioritizing while being prepared for the unexpected. Combining EFOR and IFOR representations (perspective taking) takes advantage of both the efficient removal of task-irrelevant variance and fast mental simulation. When infants start to learn by copying others’ actions (Meltzoff, 1995; Tomasello et al., 2005; Nielsen, 2006), it is important for them to hold both EFOR and IFOR representations so that imitation and emulation are possible.

## SUMMARY

The central theme in our proposal is that the complex achievements in either spatial cognition or social cognition may rely on the fundamental processes of spatio-temporal integration and, moreover, that there is a set of distributed brain regions shared by both types of cognition. In our framework, both spatial and social abilities arise in the form of spatio-temporal association in which the mind constantly deals with the temporal instability in the environment by predictive learning. In the effort of extracting statistical regularities, the internal representations evolve by first partitioning the environmental variances – namely, developing FOR-based representations – then, encoding statistical invariance at different levels of abstractions. Since the statistical regularities include not only the spatial relations of static configurations but also the temporal relations between sequential events, predictive learning links various representations with different anchors (spatial integration) at different time points (temporal integration). Together, abstract knowledge of the environment (including those about other’s beliefs and intentions) emerges from the expectation-driven competitions among multiple FOR-based representations.

In our view, different abilities are not domain-specific *per se*, rather, they are subject to the competing demands of computational efficiency and flexibility, yet are bounded by the statistical structures in the environment. By reinterpreting the results from the two-cannon experiment (Tamborello et al., 2012) and the false-belief task (Onishi and Baillargeon, 2005) and reviewing recent neurocognitive findings, we advocate an integrated approach that connects low-level perceptual processes, such as spatial representations, with high-level functions such as belief reasoning. The advantage of this approach is that, rather than singling out a certain brain system for a certain set of cognitive abilities (e.g., the TPJ for belief reasoning), we can pursue a better understanding of the mind–environment interaction over a developmental continuum. For example, the FOR-based account proposed here largely relies on the mechanisms of attentional network in spatial cognition, which have been extensively studied on from non-human animals to human infants and adults (for reviews, see, Corbetta and Shulman, 2002; Posner et al., 2006; Corbetta et al., 2008; Kavšek, 2013). Thus, this account may provide not only a transparent partitioning of the environmental statistics, but also potential explanations for the relationship between different abilities and the development of specific attentional networks. For instance, it has been suggested that “rudimentary executive attention capacities may emerge during the first year of life but that more advanced conflict resolution capacities are not present until 2 years of age” (Posner et al., 2006, p. 1425). This line of reasoning could explain why young infants suddenly appear to comprehend the complex world and pass various spatial tasks (McCrink and Wynn, 2007; Surian et al., 2007; Kovács et al., 2010; Gweon and Schulz, 2011; Téglás et al., 2011).

Legend has it that in formulating his theory of gravitation, Newton was inspired by observing the acceleration of an apple falling from a tree. Subsequently, he inferred the existence of gravity and extended the effect from to the top of the tree to the Moon (White, 1991). Perhaps more interestingly, Newton also first stated the principle of relativity (later modified by Einstein), which essentially claims that observations of the physical world depend on the particular “frame of reference” (Feynman et al., 1963, p. 162). Although we may never know the exact details of his revelation, the “apple incident” exemplifies how early perceptual analyses are triggered by temporal instability in the environment and the resulting extraction of statistical regularities with various reference points. In addition, it illuminates recent proposals that complex achievements such as mathematics and geometry, which are uniquely human in their full linguistic and symbolic realization, rest nevertheless on a set of core knowledge systems that are driven by the representations of object, space, time and number (Spelke and Kinzler, 2007; Spelke et al., 2010), and, knowledge structures emerge from non-cognitive processes by dynamic associations (McClelland et al., 2010). While controversies still exist between seemingly diverging perspectives, we take the primary theme of the debates to be the converging efforts of seeking for the cognitive or non-cognitive origins of human thinking and reasoning abilities. If we subscribe to the notion of “bounded rationality” (Simon, 1982), both spatial and social abilities are bounded by the learning agent’s computation capacity and the structure of the environment. In order to bridge the conceptual gaps between spatial and social cognition, the key is to understand the interactions between “genetic endowment and the environment” (Ruffman and Perner, 2005, p. 462).

## Conflict of Interest Statement

The authors declare that the research was conducted in the absence of any commercial or financial relationships that could be construed as a potential conflict of interest.
